# Assessment of water, sanitation and hygiene practices for prevention and control of COVID-19 in Kenya

**DOI:** 10.1093/inthealth/ihab077

**Published:** 2021-12-02

**Authors:** J Mwai, D Nyole, M Abdi, I Ahmed, J Mutai, L Kaduka, P Ndemwa, J Omogi

**Affiliations:** Centre for Public Health Research, Kenya Medical Research Institute, Kenya; Moi University; Centre for Public Health Research, Kenya Medical Research Institute, Kenya; Centre for Public Health Research, Kenya Medical Research Institute, Kenya; Centre for Public Health Research, Kenya Medical Research Institute, Kenya; Centre for Public Health Research, Kenya Medical Research Institute, Kenya; Centre for Public Health Research, Kenya Medical Research Institute, Kenya; Jomo Kenyatta University of Agriculture and Technology

**Keywords:** control, COVID-19, prevention, Kenya, WASH

## Abstract

**Background:**

Safely managed water, sanitation and hygiene (WASH) services are an essential part of preventing and protecting human health during infectious disease outbreaks, including the current coronavirus disease 2019 (COVID-19) pandemic. Additionally, adherence to COVID-19 measures, including washing hands using soap and proper waste disposal, no doubt can improve containment of the virus.

**Methods:**

A cross-sectional survey was conducted in Kilifi and Mombasa Counties in Kenya. A total of 612 quantitative data were collected using a mobile data collection tool Open Data Kit. Parametric and non-parametric tests were used to examine factors associated with WASH practices and control of COVID-19 in Kenya.

**Results:**

More than half of the respondents were from Kilifi, 431 (70.4%) were female and the mean age was 38.2±14.8 y. Households in Kilifi were most likely not to have enough water, while Mombasa households were more likely to pay for water. Sanitation coverage was 47.6%, with more than half sharing sanitation facilities. Sharing of latrines was significantly associated with county and income level. Accessing soap was worse compared with the month prior to the survey, only 3.9% had their garbage collected by formal service providers and only 17% reported wearing any protective gear while handling waste at home.

**Conclusions:**

Water is disproportionately available in the two counties, with low sanitation coverage. There is low knowledge on hand washing and inadequate waste disposal services.

## Introduction

Coronavirus disease 2019 (COVID-19) is an infectious disease caused by the novel severe acute respiratory syndrome coronavirus 2 (SARS-CoV-2). The disease has presented an unprecedented global threat to human health and economies since the first case was declared in Wuhan, China in December 2019.^1,2^ In March 2020, the World Health Organization (WHO) declared it a pandemic. With no effective treatment or vaccine available then, mitigation and containment measures were adopted across the world to prevent and control the spread of the virus.^3^

**Figure 1.**
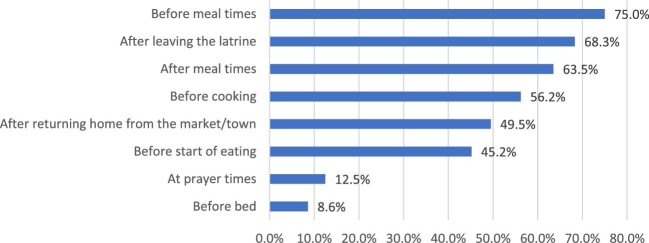
Times when household members wash their hands.

The world's poorest received the COVID-19 shock on top of existing major urban water and sanitation service deficits, pointing towards a potentially overwhelming burden to contain the virus. Low access to, reliability and quality of water, sanitation and hygiene (WASH) present risks in developing countries, Kenya included. Large cities like Mombasa also face risks stemming from population density and informal settlements.^4^

Provision of water and sanitation plays an essential role when it comes to protecting human health during all outbreaks. According to the WHO, consistent application of WASH practices, both in healthcare settings and the community, are key in preventing person-to-person transmission of COVID-19 and many other infectious diseases. Handwashing may inactivate organisms, but this is not always necessary to prevent disease transmission.^5^ Hand hygiene can be achieved through washing with adequate quantities of clean water and soap or rubbing hands with an alcohol-based solution. The recommended time for a proper handwashing is said to be at least 20 s.^6^

Experts have pointed out that urban settings have disruptive factors, including inequalities exacerbated by the rapid influx of people. This has resulted in insufficient supplies of fresh water, poor sanitation facilities and ineffective ventilation systems, increasing the risk of outbreaks. Weaknesses in access and utilization of WASH put millions of lives at greater risk of COVID-19.^7^ According to the WASH joint monitoring programme report, only 59% of Kenyans have access to basic water services and only 29% have access to sanitary services. With only 14% of Kenyans in rural and informal settlements having access to hand washing and soap facilities at home during the pre-COVID period, this situation may have been exacerbated by the pandemic. In addition, the situation may have been made worse with measures such as lockdowns and curfews.^8,9^ Ensuring availability and access to water and sanitation is therefore critical if COVID-19 is to be controlled in Kenya and other sub-Saharan countries.

This study assessed WASH services and practices in Mombasa and Kilifi Counties. It also assessed the knowledge and practices towards COVID-19 among residents during the pandemic.

Water in Mombasa County is managed by the Mombasa Water and Sewerage Company, with sources including 452 shallow wells spread across the entire county, 3 permanent springs, 4 water pans and a number of boreholes operated by private investors, non-governmental organizations and local community-based organizations. According to the Mombasa County Integrated Development Plan of 2018, the sanitation coverage in the county stands at 71%.^10^

In contrast, Kilifi County toilet coverage is estimated at 67% and 30% of households have hand washing facilities. A significant proportion of the population in the county has no access to basic sanitation facilities, posing serious public health implications. More importantly, the proportion of households with access to sanitation facilities varies across and between major urban centres and peri-urban areas and the concentration of these facilities tends to decline towards the rural areas within the county.^11^

## Methods

### Study setting

This was a community-based cross-sectional survey that adopted a convergent mixed method design. The study was conducted in Kilifi and Mombasa Counties in the coastal region of Kenya. According to the Kenya National Census of 2019, the two counties had a combined population of 2 662 120.^12^ The survey was conducted between 25 November and 3 December 2020, during the second wave of the COVID-19 pandemic in Kenya.

### Sample size determination and sampling procedure

An estimation of 58% based on the prevalence of WASH coverage in Kenya was used to determine the sample size. The acceptable error rate and significance level were set at 3% and 0.05, respectively. Using a simple random sampling formula, we calculated a preliminary sample size of 374. A design effect of 1.5 was used to further adjust the sample size to include a non-response rate of 5%, giving a sample size of 612.

A multistage sampling technique was used to select the villages included in the study. In the first stage, subcounties were selected using random sampling in selecting three subcounties from a total of six subcounties in Mombasa, while in Kilifi, all seven subcounties were included in the study. The second stage involved ward selection, in which a list of all wards identified from the 10 subcounties, totalling 52, were systematically sampled, giving 14, with 3 acting as the kth. Finally, to select the specific villages, proportional stratified random sampling in relation to the population size was used and a total of 28 villages were sampled.

In Mombasa County, the study was conducted in three subcounties: Changamwe, Kisauni and Mvita. In Kilifi County, the study was conducted in seven subcounties: Magarini, Kilifi South, Ganze Kaloleni, Rabai, Malindi and Kilifi North.

### Study population

The study targeted household heads (men and women), 18–60 y of age, residing in the two counties. In each village, with the assistance of community health volunteers or village elders, households were randomly selected. Within the households, a kish grid was used to select the households.^13^

### Data collection

A structured questionnaire converted into the Open Data Kit was used to collect demographic data; household knowledge, attitudes and practices of COVID-19; and hand hygiene and WASH practices. Data were saved after finishing each household interview and could not be edited further. This was meant to protect the integrity of the data from the households. Data were uploaded to a secure server and daily summary reports were produced to evaluate daily targets and completeness of data collection.

### Data analysis

Data was analysed using descriptive statistics and presented in tables and figures, summarizing the WASH situation in the two counties. Univariate analysis was conducted for all variables to compare outcomes of interest. Proportions were used for categorical variables and measures of central tendency and dispersion were for continuous variables. Using the Shapiro–Wilk test, knowledge, practice and perception responses were tested for normality before analysis. The χ^2^ and Fisher’s exact test were used to determine significant differences in the key outcome variables.

Significant levels were set at α=0.05. Logistic regression was done to determine the predicting factors associated with WASH practices.

### COVID-19 measures and protocol adherence

Prior to conducting the research, the county governments of Kilifi and Mombasa and the research team held an online meeting and discussed the best way to prevent COVID-19 infections while conducting the research. Among the requirements were to extend the number of days and work with a small number of research assistants. A total of three vans were made available in order to maintain physical distancing during travel. All the research assistants were given masks and were advised to wear them correctly at all times; they were also provided with hand sanitizer. The team agreed to rest anyone who developed COVID-19 symptoms. In addition, the research team from the Kenya Medical Research Institute underwent COVID-19 testing 24 h prior to travelling to the field.

## Results

### Demographic and socio-economic characteristics

We interviewed 612 household heads (Kilifi, 55.4%; Mombasa, 44.6%). The respondents were mostly women (431 [70.4%]), with 329 (53.7%) households headed by adult males. The mean age of the household heads was 38.2±14.8 y. Most (438 [71.6%]) of the respondents had a primary education, but 126 (20.6%) had no formal education. A third (38%) of the household heads indicated that they were unemployed at the time of the interview as shown in Table 1, with 40% of the households earning <${\$}$30 per month. A total of 87.6% of households owned a mobile phone, 52.2% owned a radio and 31.3% owned a television and 48.4% of the houses had electricity. Only 28.2% had solar panels, 12.5% had a clock, 2.5% owned a refrigerator and 0.5% had a fixed telephone.

### WASH practices

The main water sources for the households were piped water (269 [43.9%]), public water kiosks (147 [24%]) and boreholes (122 [19.9%]), while 70 (11.4%) of the households got their water from streams, rivers or ponds. A total of 365 (59.6%) households indicated that the water they got was enough to satisfy their needs, although 82.5% of the households had to pay for the water from water vendors.

As shown in Table 2, there was a significant difference (χ^2^=7.19, p<0.05, odds ratio [OR] 0.6) in availability of water by counties. The odds of Kilifi residents having enough water was 1.7 times lower compared with the residents of Mombasa County, suggesting that residents in Mombasa County had better access to water compared with those in Kilifi. Additionally, there was a strong significance (χ^2^=23.7, p<0.05, OR 0.4) between payment for water and the county, with the odds of residents in Kilifi paying for water being 2.2 times lower compared with residents in Mombasa. This implies that although residents in Mombasa were more likely to access water than those in Kilifi, were also more likely to pay.

**Table 1.** Sociodemographic characteristics (N=612)

Variable	n	%
County		
Kilifi	339	55.4
Mombasa	273	44.6
Household head		
Adult female	218	35.6
Adult male	329	53.8
Elderly female	24	3.9
Elderly male	41	6.7
Gender		
Male	181	29.6
Female	431	70.4
Age (years), mean	38.2±14.8	
Age group (years)		
18–27	165	27
28–37	178	29.1
38–47	118	19.2
48–57	67	11
58–67	54	8.8
>67	30	4.9
Marital status		
Married	457	74.6
Single	106	17.3
Widowed	35	5.7
Divorced	14	2.3
Education level		
None	126	20.6
Primary	438	71.6
Secondary	48	7.8
Main form of employment		
Self-employed	134	21.9
Farmer	139	22.7
Employed	131	21.4
Unemployed	208	34

**Table 2.** Association between sociodemographic and water-related indicators

	Source of water			Availability of enough water			Payment for water		
Variables	Unimproved source, n (%)	Improved source, n (%)	Test statistics	Yes, n (%)	No, n (%)	Test statistics	Yes, n (%)	No, n (%)	Test statistics
County									
Kilifi	45 (13.2)	294 (86.7)	χ^2^=2.5, p=0.11	186 (54.8)	153 (45.3)	χ^2^=7.19, p=0.007, p=0.64, (0.45–0.89)	257 (75.8)	82 (24.1)	χ^2^=25.0, p=0.000, p=0.32, (0.20–0.51)
Mombasa	25 (19.2)	248 (90.8)		179 (65.6)	94 (34.4)		248 (90.8)	25 (9.2)	
Gender									
Female	52 (12.1)	379 (87.9)	χ^2^=0.56, p=0.45	259 (60.1)	172 (39.9)	χ^2^=0.12, p=0.72	356 (82.6)	75 (17.4)	χ^2^=0.007, p=0.93
Male	18 (9.9)	163 (90.1)		106 (58.6)	75 (1.4)		149 (82.3)	32 (17.7)	
Marital status									
Married	61 (13.3)	396 (86.6)	Fisher's exact test, 0.43	266 (58.2)	191 (41.8)	χ^2^=3.5, p=0.319	367 (80.3)	90 (19.7)	Fisher's exact test, 0.06
Divorced	5 (14.3)	30 (85.7)		25 (71.4))	10 (28.6)		9 (25.7)	26 (74.3)	
Single	4 (3.8)	102 (96.2)		67 (63.2)	39 (36.8)		8 (7.5)	98 (92.4)	
Widowed	0	14 (100)		7 (50)	7 (50) (34)		0	14 (100)	
Education level									
None	29 (23)	97 (77)	Fisher's exact test, 0.32	78 (61.9)	48 (38.1)	χ^2^=0.86, p=0.64	95 (75.4)	31 (24.6)	χ^2^=5.6, p=0.06
Primary	39 (8.9)	399 (91.1)		261 (59.5)	177 (40.4)		370 (84.5)	68 (15.5)	
Secondary	2 (4.1)	46 (95.8)		26 (54.2)	22 (45.8)		40 (83.3)	8 (16.7)	
Age group (years)						χ^2^=7.8, p=0.17			
18–27	18 (21.2)	67 (78.8)	Fisher's exact test, 0.09	108 (65.4)	57 (34.5)		141 (85.5)	24 (14.5)	χ^2^=4.89, p=0.43
28–37	16 (15.4)	88 (84.6)		97 (54.5)	81 (45.5)		151 (84.8)	27 (15.2)	
38–47	15 (22.1)	53 (77.9)		65 (55.1)	53 (44.9)		95 (80.5)	23 (19.5)	
48–57	8 (21.6)	29 (73.4)		42 (62.7)	25 (37.3)		52 (77.6)	15 (23.4)	
58–67	9 (32.1)	19 (67.9)		37 (68.5)	17 (31.5)		44 (81.5)	10 (18.5)	
>67	4 (19)	17 (81)		16 (53.3)	14 (46.7)		22 (73.3)	8 (26.7)	
Income (Kenyan shilling)									
<2999	24 (19.5)	99 (80.5)	Fisher's exact test, 0.078	124 (53.5)	108 (46.5)	χ^2^=19.0, p=0.002	196 (84.5)	36 (15.5)	Fisher's exact test, 0.06
3000–5999	29 (29.9)	68 (70.1)		88 (54.7)	73 (45.3)		122 (75.8)	39 (24.2)	
6000–8999	8 (14.8)	46 (85.2)		57 (65.5)	30 (34.5)		73 (83.9)	14 (16.1)	
9000–11 999	4 (21)	15 (79)		33 (73.3)	12 (26.7)		37 (82.2)	8 (17.8)	
12000–14 999	1 (5)	19 (95)		27 (84.4)	5 (15.6)		31 (96.9)	1 (3.1)	
>14 999	4 (13.3)	26 (86.7)		36 (65.5)	19 (34.5		46 (83.6)	9 (16.4)	

A total of 205 (33.5%) households reported using flush or pour toilets, 341 (55.7%) used pit latrines and 59 (9.6%) had no toilet facility. A significant proportion (290 [52.4%]) of those households with sanitation facilities reported sharing latrine facilities. The overall sanitation coverage among the households was 47.6%. Sharing of latrines was significantly associated with the county (χ^2^=90.1, p<0.05) and house ownership (χ^2^=92.3, p<0.05).

A strong statistical significance (p<0.05) was observed between sharing latrine facilities and income, with the odds of sharing a latrine among those who earned ${\$}$30–${\$}$60 being 2.1 times lower compared with those who earned <${\$}$30. Similarly, the odds of sharing a latrine among those who earned >${\$}$140 was 3.7 times lower compared with those who earned <${\$}$30. This suggests that income is a strong predictor for sharing of latrine facilities. According to the findings, households earning ${\$}$90–${\$}$120 were more likely (2.4 times higher) to have enough water compared with those earning <${\$}$30. Similarly, the odds of a household earning ${\$}$120–${\$}$140 having enough water was 4.7 times higher compared with a household earning <${\$}$30.

The findings show that the odds of Mombasa residents sharing a latrine were 5.6 times higher compared with residents in Kilifi, with a strong statistical difference (p<0.05). This suggests that a number of residents in Mombasa shared latrines. The odds of households who own a house sharing a latrine facility were 7.6 times lower compared with those who rented. Similarly, the odds of sharing a latrine for a household having one bedroom was 5.2 times lower compared with a household living in a single room. For a household having two bedrooms, the odds were 5 times lower, while those having three or more bedrooms were 33.3 times lower. This suggests the likelihood of not sharing a latrine facility as the number of bedrooms increases. This clearly shows the effects of socio-economic characteristics on WASH status.

Although 396 (64.7%) reported challenges in accessing soap, 594 (97%) households indicated that they practice hand washing. Topping the list of the challenges was that there are other priorities (62.4%) and soap was too expensive (57.2%). A total of 321 (54.3%) households reported that they wash their hands 3–5 times a day, compared with 46 (7.8%) who never wash their hands. In terms of when they wash their hands, 75% of households mostly wash their hands before meal times, after leaving the latrine (68.3%), after meal times (63.5%) and before cooking (56.2%) as shown in Figure 1. According to 34.6% of the households, accessing soap at the time of the study was more difficult compared with a month prior to the survey, while 32.8% indicated that access to soap was easier at the time of the survey.

Almost half (49.1%) of the households reported burying or burning their garbage, 32.1% disposed it within the household yard or plot while only 3.9% had their garbage collected by formal service providers. Only 104 (17%) of the participants reported wearing any protective gear while handling waste at home.

Households reported receiving information and education on issues related to WASH mostly at health facilities or from community health volunteers. The information received was on water use (53.6%), hygiene (42.6%), hand washing (39.2%) and the use of soap (33.3%). Hand washing with soap (91.7%), the use of alcohol-based hand sanitizer (43.3%) and safe storage of household water (41.5%) were the information most received by households on water and sanitation.

## Discussion

WASH services play a critical role in the prevention and control of SARS-CoV-2 transmission.^14^ To the best of our knowledge, this is the first community-based survey done to assess WASH practices in Kenya during the COVID-19 pandemic. Currently there is limited information on availability of WASH services and practices during COVID-19, especially in rural areas and urban slums.

Kenya is a water-scarce country, with only 59% of the population having access to clean water. This coverage is much lower in informal settlements and rural areas.^8^ In areas with water, the supply varies and prices fluctuate based on availability. Inequalities in access and availability of water tend to affect water-related behaviour such as hand washing. During pandemics like COVID-19, water is used in important activities such as hand washing, maintaining a clean environment and sanitizing surfaces as recommended for COVID-19 infection control.^8,12,13^

A majority of the respondents in this study were living in informal settlements and rural areas with the main sources of water for most households being piped water, public kiosks and boreholes. Six of ten households indicated that the water supply was sufficient for their needs. Also,

82.5% had to pay for water, with the average cost per 20-litre being 20 Kenya shillings (US${\$}$0.44). The fact that Kilifi households were unable to access enough water while Mombasa households were more likely to pay for water shows the inequality of the availability of this important basic need. This implies that there is a need for provision of an affordable, quality water supply to the population. However, this contradicts the information given by the authorities in the two counties that they offer an uninterrupted supply of water to the residents, regardless of payments. Previous studies indicate that only 40% of Kilifi residents have access to piped water, compared with Mombasa where <25% of residents access piped water and the remaining gaps are filled by private providers through public kiosks and boreholes.

This has led to many residents in both counties who pay to access water.^14^ This implies that both Mombasa and Kilifi Counties need to provide mechanisms for residents to access quality, affordable water.

According to the WHO, hand washing with soap and water is one of the key infection control measures to prevent COVID-19. Globally, 3 billion people and approximately 75% of sub-Saharan countries lack basic home hand washing facilities with water and soap. In contrast, in urban areas <24% of the population has access to hand hygiene facilities.^12,15^ This study found the prevalence of reported hand washing with water to be high (97%), while the prevalence of those who reported hand washing with soap and water was only 58%. This could be attributed to the health promotion activities that have been done by the Kenyan government on the importance of hand hygiene as a COVID-19 infection control measure. Studies done in the Philippines and Vietnam had similar findings, with high rates of handwashing being reported in those countries.^16–18^ Approximately 54.3% of the respondents reported that they wash their hands at least 3–5 times a day. Some of the critical times for hand washing included before eating food, after using the toilet and before cooking. Few reported washing hands when coming back to their homes or removing their mask, as required. Most of the respondents experienced challenges in accessing soap, especially during the COVID-19 period. This could be linked to the economic status of the respondents and the need for other competing priorities like water and food vs purchasing soap. There is therefore a need for counties to put in place a mechanism for providing soap in public places to ensure effective hand washing is done and to promote behavioural change towards frequent hand hygiene, especially for prevention of COVID-19.

Our findings show that sanitation coverage was remarkably high, with 90% coverage of access to a latrine or pit latrine. More than half of the respondents reported sharing latrines/toilets, with households in Kilifi more likely not to share toilets compared with those in Mombasa. This could be attributed to the rural setting in Kilifi compared with Mombasa. According to our findings, the overall access to sanitation facilities was 47.6% compared with the national figure of 29%.^6^ The low numbers of open defecation could be due to the ongoing Ministry of Health interventions in conjunction with United Nations Children's Fund, the aim of which is to have no open defecation. The proportion of households sharing toilets/latrines in this study was higher than the Kenyan estimate of 44%.^15^

In terms of waste disposal, most households practiced inappropriate methods of waste disposal, with <20% reporting wearing any protective gear while handling waste at home.

The mandatory use of masks to prevent the spread of COVID-19 has seen the introduction of disposed masks to household waste.^16^ Literature shows that waste generated by the COVID-19 outbreak has posed a major environmental and health concern in many countries. In particular, inadequate solid waste management may increase the spread of coronavirus, especially in developing countries.^17^

Community health volunteers and health facilities were the main sources of information and education on issues related to WASH. This highlights the vital role that community health volunteers play in providing health education, especially for COVID-19 prevention through WASH. The findings revealed a high level of awareness of COVID-19 and knowledge on preventive measures, with radio and television being the main sources of information on COVID-19. The findings contrast with a study in the Middle East where the main source of information was doctors and other medical staff, followed by social media. However, it is similar to a study that found newspapers to be the least used source of information.^18^ There is a need to leverage existing platforms such as community meetings, radio and television to disseminate information and educate communities on proper WASH practices.

## Conclusions and recommendations

Disproportionate water provision was clear in the two counties with residents of Kilifi being the most affected.

More than half of the residents in both counties still share latrine facilities. There is a need for the education of all the stakeholders, including tenants, landlords and community leaders, on the proper cleaning of shared facilities. The interventions should be co-designed with the users and the county governments should institute policies and regulations regarding these practices.

A low level of knowledge exists on hand washing after removal of masks or having come from outside the house. More awareness and behavioural changes on the importance of hygiene need to be carried out by all stakeholders, led by the county governments. Less than a third of residents reported best practices on waste disposal, hence the need to improve waste collection. There is also a need for more effective messaging by the county governments and other stakeholders and to promote behavioural changes towards waste disposal.

## Data Availability

The data underlying this article will be shared on reasonable request to the corresponding author.
